# Supplementary material for: Rapid turnover of life-cycle-related genes in the brown algae
Source: Genome Biol. 2019 Feb 14;20:35. doi: 10.1186/s13059-019-1630-6 (PMC6374913; doi:10.1186/s13059-019-1630-6)
Supplement: Supplementary file 3 — Protein alignments, corresponding Gblocks html file and CDS sequences for the lineage conserved gametophyte-biased genes. (ZIP 2109 kb) [file 13059_2019_1630_MOESM3_ESM.zip › Con_GA_alignments/Ec-05_005050.aa_ali.fasta-gb.htm]

Ec-05\_005050.aa.fasta.trans.aa\_ali.fasta


## Gblocks 0.91b Results

Processed file: **Ec-05\_005050.aa.fasta.trans.aa\_ali.fasta**  
Number of sequences: **2**  
Alignment assumed to be: **Protein**  
New number of positions: **370** (selected positions are underlined in blue)

```
                         10        20        30        40        50        60
                 =========+=========+=========+=========+=========+=========+
Ec-05_005050     MYQSAAHTAIEAQAECFGKPLIRRELAGRAVNQALQYSLTEDDEVEDLFLLLKEVKERFP
MSTRG.14169.1    ----------------------------------------------------------FP
                                                                           ##


                         70        80        90       100       110       120
                 =========+=========+=========+=========+=========+=========+
Ec-05_005050     EVEGVGSGAILSNYQRTRVENVCGRLGLTSLAYLWRRPQSPLLAEMVEAGLNAVLVKVAS
MSTRG.14169.1    EVEGVGSGAILSNYQRTRVENVCGRLGLQSLAYLWRRPQSPLLAEMVEAGLDAVLVKVAS
                 ############################################################


                        130       140       150       160       170       180
                 =========+=========+=========+=========+=========+=========+
Ec-05_005050     FGLEPSKHLGRSLARLRPFFESLHSRCGFHVCGEGGEYETLTLDCPLFVRKLVLDETVVV
MSTRG.14169.1    FGLDPSKHLGRSLARLRPFFESLHSRCGFHVCGEGGEYETLTLDCPLFVRKLVLDETVVV
                 ############################################################


                        190       200       210       220       230       240
                 =========+=========+=========+=========+=========+=========+
Ec-05_005050     NHSDDMFAPVGLLRVVKCHTEPKTPP---LTGGSISAADN--------------------
MSTRG.14169.1    NHSDDMFAPVGLLRVVKCHTEAKAPLGTSASTGSGSTHDSNNPAVSEAASPPADAGDDSK
                 #########################                                   


                        250       260       270       280       290       300
                 =========+=========+=========+=========+=========+=========+
Ec-05_005050     ---NTG--------------KDN-------------ATGGT-------------------
MSTRG.14169.1    SGLNRGKAGCEVDPQAAPEGMSDVRAPSSASAAETEAVASTAAPSAAAATSCAPAAAAAP
                                                                             


                        310       320       330       340       350       360
                 =========+=========+=========+=========+=========+=========+
Ec-05_005050     AL----------STAEGSGGLVYLSGVR--AAARWPGAGGGAAAAAAAARQAADCLEAAR
MSTRG.14169.1    ASSPPPPPSCLPHVHEGSGGLVYVSGVCGSSSTRRGTGGAEAAGATAAAEQAADCLAAVR
                                ############      ###########################


                        370       380       390       400       410       420
                 =========+=========+=========+=========+=========+=========+
Ec-05_005050     TGLEGIGVCLRDVCFVHLYLRDMRLFSAVNEEYCKWFEGENPPSRSCVSVPLPLGCSVML
MSTRG.14169.1    RGLEGVGVSFRDVCFVHLYLRDMGHFSTINEEYCKWFQGENPPSRSCVSVPLPSGCSVML
                 ############################################################


                        430       440       450       460       470       480
                 =========+=========+=========+=========+=========+=========+
Ec-05_005050     DLVALRGSGEALAKEGAACAQRQVLHVRSISSWAPVCIGPYCQANTLGPGGGFALVAGQI
MSTRG.14169.1    DVVALRGSGEALAKEGAACAHRQVLHVRSISSWAPVCIGPYCQANTLGPGGGIALIAGQI
                 ############################################################


                        490       500       510       520       530       540
                 =========+=========+=========+=========+=========+=========+
Ec-05_005050     GLQAASMTFPARRSGRPPVPCAGGATSKEAISERRPIHEQELSLCVSHASSVASAVGASV
MSTRG.14169.1    GLQPETMNFPARTPLCPRSPATAHLPSASDVG-ALPIHERELSLCVSHASSVASAVGASV
                 ###########################        #########################


                        550       560       570       580       590       600
                 =========+=========+=========+=========+=========+=========+
Ec-05_005050     SRGCVFATLYVSEEAAAAAAAAAAGGGAAVAAHAKAGAMDRTDEEGAVAGEGRGDTRRTS
MSTRG.14169.1    SRGCVFATLYVSQ-----------------------------------------------
                 ############                                                


                        610       620       630       640       650       660
                 =========+=========+=========+=========+=========+=========+
Ec-05_005050     SSDAGTSAGAAAPAGGSGGCLEGMVDECRVLMEGRLSQEQAEAAAEAAGTEGGEDGDSAE
MSTRG.14169.1    ------------------------------------------------------------
                                                                             


                        670       680       690       700       710       720
                 =========+=========+=========+=========+=========+=========+
Ec-05_005050     DGWTSDPEELAKEAPRKMVLARRVPILAVVVPSLPRNAAVELELEQAMSLVLAGVSDAVS
MSTRG.14169.1    ------------------------------------------------------------
                                                                             


                        730       740       750       760       770       780
                 =========+=========+=========+=========+=========+=========+
Ec-05_005050     AARLRWHDVANLRVYYNARRHGPSGVAATGTGGGGGYSGGSDGIEEGFLKRATFLALAGA
MSTRG.14169.1    ------------------------------------------------------------
                                                                             


                        790       800       810       820
                 =========+=========+=========+=========+===
Ec-05_005050     TRERPAVSFVPVNGLGGGAAVSVHATAWSLDRLRTELWVRGAT
MSTRG.14169.1    -------------------------------------------
```

```
Parameters used
Minimum Number Of Sequences For A Conserved Position: 2
Minimum Number Of Sequences For A Flanking Position: 2
Maximum Number Of Contiguous Nonconserved Positions: 8
Minimum Length Of A Block: 10
Allowed Gap Positions: None
Use Similarity Matrices: Yes
```

```
Flank positions of the 4 selected block(s)
Flanks: [59  205]  [316  327]  [334  507]  [516  552]  

New number of positions in Ec-05_005050.aa.fasta.trans.aa_ali.fasta-gb:  370  (44% of the original 823 positions)
```
